# Evaluation of Wide Corneal Epithelial Remodeling after Small Incision Lenticule Extraction (SMILE) with Wide-Field Optical Coherence Tomography

**DOI:** 10.1155/2022/8764103

**Published:** 2022-03-20

**Authors:** Yiming Ye, Pei Chen, Na Yu, Linxi Wan, Min Lan, Hua Zheng, Keming Yu

**Affiliations:** State Key Laboratory of Ophthalmology, Zhongshan Ophthalmic Center, Sun Yat-sen University, Guangdong Provincial Key Laboratory of Ophthalmology and Visual Science, Guangdong Provincial Clinical Research Center for Ocular Diseases, Guangzhou, China

## Abstract

**Purpose:**

This study aims to assess the corneal epithelial remodeling within a 9 mm diameter zone induced by small incision lenticule extraction (SMILE) and evaluate its relationship with the refractive outcomes.

**Methods:**

A total of 64 eyes of 64 patients were included. Wide-field optical coherence tomography (OCT) was used to measure the epithelial thickness (ET) across a 9 mm diameter area, preoperatively, and after one day, one week, one month, three months, and six months postoperatively. The ET changes were compared among the different time points and analyzed zones.

**Results:**

The ET increases from one week to three months and stabilized from three months to six months. Compared to the preoperative values, the mean ET changes at six months in central (2 mm), paracentral (2–5 mm), mid-peripheral (5–7 mm), and peripheral (7–9 mm) zones were 4.37, 4.36, 1.61, and −1.59 *μ*m, respectively. The correlation between the epithelial thickening and the amount of myopia correction was positive in central (*P* = 0.001) and paracentral zones (*P* < 0.001) and negative in peripheral zone (*P* = 0.006). The intended diameter of the optical zone was negatively related to epithelial hyperplasia in the central (*P* = 0.020) and paracentral zone (*P* = 0.006), and the correlation was positive in the mid-peripheral zone (*P* = 0.001). The epithelial thickening of central zone (*P* = 0.012) and the difference of mean ET between central and paracentral zone (*P* = 0.020) were negatively related to the spherical equivalent at six months.

**Conclusion:**

An asymmetric lenticule-like pattern of epithelial remodeling occurred in 9 mm diameter cornea at six months after myopic SMILE. The epithelial remodeling may affect the refractive outcomes of SMILE.

## 1. Introduction

Small incision lenticule extraction (SMILE) is an all-in-one, flap-free femtosecond laser surgery that has been introduced for more than 10 years [1]. The efficacy, safety and predictability of SMILE have been promising for correcting myopia and myopic astigmatism [2–4]. In SMILE, the intrastromal lenticule created by femtosecond laser is removed through a small incision of 2 mm in length. Compared to other corneal refractive surgeries, this unique procedure preserves most of the epithelium, Bowman's layer, and anterior stroma and leads to less disruption on the corneal surface and biomechanics [5, 6]. It is well established that corneal epithelial remodeling occurs after corneal refractive surgery. The central flattened stroma results in corneal epithelial thickening to restore the smooth optical surface [7–10]. Various studies have reported the pattern of corneal epithelial remodeling following LASIK and PRK, and central corneal epithelial hyperplasia was even shown to be a potential cause of postoperative regression [11–14]. However, few studies have evaluated the epithelial thickness (ET) changes following SMILE. Furthermore, most of these studies measured the ET with conventional spectral-domain OCT, providing an ET mapping with an analysis diameter of merely 6 mm [10, 15, 16]. The ET changes of the peripheral cornea following SMILE are not fully elucidated. An ET map of the entire cornea would be helpful to elucidate the impact of epithelial remodeling on clinical outcomes.

Recently, the new wide-field RTVue-XR OCT system has become available for measuring the corneal ET and stromal thickness up to a 9 mm diameter zone. This novel device has been reported to have excellent repeatability and reproducibility for 9 mm diameter ET and corneal thickness measurements in both normal corneas and corneas with different conditions, including keratoconus, dry eyes, and contact lens wear and after FS-LASIK or PRK [17–20]. A characteristic epithelial remodeling after SMILE for high myopic astigmatism correction was also reported in our previous study with this OCT [21]. However, the relationship between the ET changes, the treatment parameters, and the impact of the epithelial remodeling on the refractive outcomes of SMILE still needs in-depth investigation.

The present study aimed to observe the corneal epithelial remodeling across a 9 mm diameter cornea following SMILE using the wide-field OCT and evaluate its potential effect on the refractive outcomes.

## 2. Patients and Methods

### 2.1. Patients

This prospective observational study included 64 eyes of 64 patients who underwent SMILE for treatment of myopia or myopic astigmatism at Zhongshan Ophthalmic Center (ZOC) between September and December 2017. The current study was approved by the Medical Ethics Committee of ZOC (2017KYPJ087) and adhered to the tenets of the Declaration of Helsinki. Written informed consent was obtained from each patient before the surgery. The inclusion criteria were as follows: stable myopia for more than two years, a CDVA of 20/25 or better, a central corneal thickness of no less than 480 um, and a calculated postoperative residual stromal bed of more than 280 um. The exclusion criteria were as follows: a long history of contact lens wear, moderate-to-severe dry eyes (Schirmer I < 10), and a history of other ocular diseases, such as keratoconus, corneal opacity, corneal dystrophy, or ocular surgery. All patients underwent a complete clinical examination, including medical history, noncontact tonometry, Scheimpflug tomography with Pentacam HR (Oculus Optikgerate, Wetzlar, Germany), Sirius system combining Placido disk topography with Scheimpflug tomography (CSO, Florence, Italy), ultrasound pachymetry (AL-1000; TOMEY, Nagoya, Japan), dry eye assessment (Schirmer I), tear film breakup time assessment, slit-lamp biomicroscopy, dilated fundus examination, uncorrected (UDVA) and corrected distance visual acuity (CDVA), and manifest and cycloplegic refraction. The 9 mm diameter epithelial thickness measurement was performed using RTVue-XR OCT (version 2017.1.0.155; Optovue, Inc., Fremont, CA).

### 2.2. Surgical Procedure and Postoperative Care

All SMILE procedures were performed under topical anesthesia by an experienced surgeon (KY) using the VisuMax 500 kHz femtosecond laser (Carl Zeiss Meditec AG, Jena, Germany). A standard surgical procedure was performed with the following parameters: laser-cut energy from 110 to 120 nJ, laser spot distance of 4.5 um, optical zone ranging from 6.0 to 6.9 mm, cap diameter from 7.0 to 7.9 mm, cap thickness of 110 um, and small incision of 2 mm in length at the 11 o'clock position. The femtosecond laser treatment was centered on the visual axis based on the data from the Pentacam HR, and the lenticule was dissected by a thin spatula and extracted through the incision. A balanced salt solution was used to wash the interface.

The postoperative medications were as follows: topical levofloxacin 0.3% eye drops (Cravit; Santen, Japan) four times a day for one week; dexamethasone 0.1% and tobramycin 0.3% eye drops (Tobradex; Alcon, Austria) four times a day for one week, followed by fluorometholone 0.1% eye drops (FML 0.1%; Allergan, USA) four times a day for three weeks; and preservative-free lacrimal substitutes four times a day for four weeks or more, when necessary.

These patients were followed up at one day, one week, one month, three months, and six months postoperatively. At each follow-up visit, UDVA, noncontact tonometry, slit-lamp examination, and OCT were performed. CDVA and manifest refraction were performed at one, three, and six months postoperatively.

### 2.3. Epithelial Thickness Measurements

Preoperatively and at each postoperative visit, corneal epithelial thickness maps of the 9 mm diameter zone were obtained using the RTVue-XR OCT system with a corneal adaptor module. All ET measurements were conducted between 2 : 00 PM and 5 : 00 PM, to minimize the impact of diurnal variation. The use of any eye drops was prohibited two hours before the examination.

The “PachymetryWide” scan mode was selected to measure the 9 mm diameter ET, following the manual. The scan unit was aligned before each examination. When both horizontal and vertical reflection stripes were simultaneously observed, the centration of the scan on the corneal apex was promised, and the scan was manually triggered. The ET maps were automatically generated and divided into 25 sectors by eight meridians and four circles. In the present study, the analyzed corneal area was further split into central (within 2 mm), paracentral (2–5 mm), mid-peripheral (5–7 mm), and peripheral (7–9 mm) zones. The same experienced investigator (YY) performed all the OCT scans. Three consecutive measurements were conducted to ensure the quality of the image, the signal strength of each scan should be more than 30, and the average value was used for the analysis.

### 2.4. Statistical Analysis

The statistical analysis was performed using SPSS V.24.0. The data were presented as mean ± standard deviation (SD). The normality of variables was assessed using the Kolmogorov–Smirnov test. The average ET of different analyzed zones and sections before surgery and at each postoperative visit were compared using the Friedman test, and the Bonferroni correction was used for post hoc analysis. The paired t-test was used to detect the difference in ET between the superior and inferior sections and between the nasal and temporal sections. Spearman's coefficient was used to determine the association between the ET changes of different zones and treatment parameters, and relationship between the ET remodeling and the refractive error of six months. *PP* value <0.05 was considered statistically significant.

## 3. Results

### 3.1. Demographics

A total of 64 myopic eyes (23 male and 41 female patients) were included in the present study. The mean age was 28.14 ± 6.38 years (range: 18–47 years ), and the mean preoperative SE was −5.76 ± 2.01 D (range: −1.25 to −9.88 D), with a mean central corneal thickness of 540.3 ± 28.4 *μ*m (range: 481–605 *μ*m). The average cylinder degree was −0.73 ± 0.61 D (range: 0 to −2.50 D). The preoperative information of all patients is presented in Table 1. The mean postoperative refractive error at six months was −0.05 ± 0.22 *D* (range: +0.75 to −0.75 *D*), with 60 of 64 eyes within ±0.50 D of the intended correction. The average postoperative CDVA was −0.13 ± 0.07 logMAR (range: −0.30 to 0.0 logMAR), and all eyes achieved a UDVA of 20/25 or better at the end of 6-month follow-up. The visual and refractive outcomes of all follow-up points are summarized in Table 2.

### 3.2. Corneal Epithelial Thickness Changes

**Figure 1:**
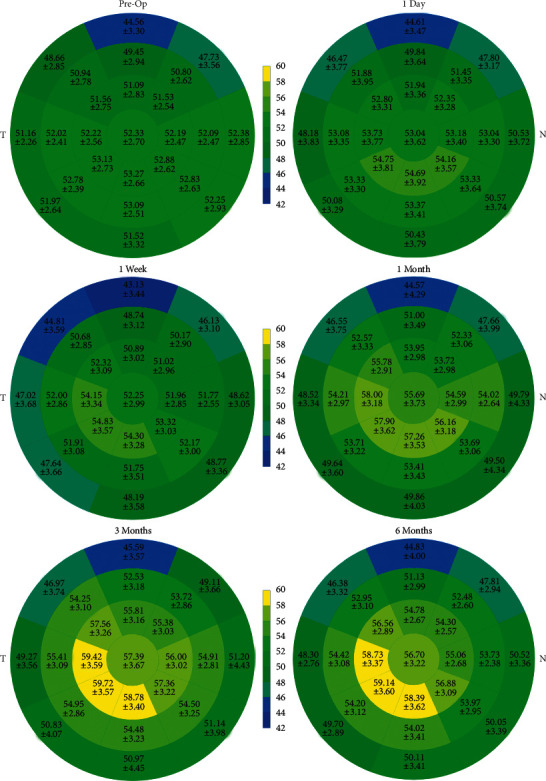
Preoperative to six-month postoperative 9 mm diameter epithelial thickness map. The corneal epithelial thickness of all 25 sections was presented by the mean and standard deviation (*n* = 64 eyes). N: nasal; T: temporal.

Figure 1 shows the longitudinal changes of average ET in 25 sections before and after SMILE. Briefly, at six months, significant epithelial thickening occurred within the 7 mm diameter cornea, and significant epithelial thinning was observed in the 7–9 mm diameter cornea, except the superior section. The average ET of central 2 mm diameter cornea increased from 52.33 ± 2.70 *μ*m (range: 47 to 60 *μ*m) preoperatively to 56.70 ± 3.22 *μ*m (range: 50 to 66 *μ*m) at the end of follow-up. The maximum ET increase of 6.51 ± 3.10 *μ*m was observed in the temporal section of the paracentral zone. The greatest epithelial thinning of 2.86 ± 2.54 *μ*m was observed in the temporal section of the peripheral zone. The average ET of the temporal section was significantly thicker than nasal section in paracentral (*P* < 0.001) and mid-peripheral zones (*P* = 0.049). However, this correspondent discrepancy was opposite in peripheral zone (*P* < 0.001). The average ET of superior sections was significantly thinner than that of inferior sections in paracentral, mid-peripheral, and peripheral zones (All: *P* < 0.001).

**Figure 2:**
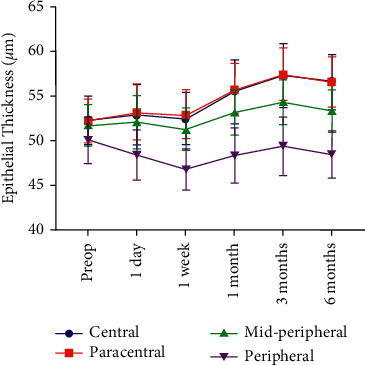
Preoperative to six-month postoperative epithelial thickness changes, in the central (2 mm), paracentral (2 to 5 mm), mid-peripheral (5 to 7 mm), and peripheral zones (7 to 9 mm). The average ET of the central, paracentral, and mid-peripheral zones significantly increased from one week to three months and stabilized from three months and six months. The average ET of peripheral zone decreased at one day, one week, and six months and underwent an increase from one week to three months (*n* = 64 eyes).

The longitudinal ET changes of all analyzed zones are shown in Figure 2. During the first one week postoperatively, a significant decrease in average ET was observed in the peripheral zones (*P* < 0.001). The average ET of the central, paracentral, and mid-peripheral zones was unaltered compared to the preoperative values. From one week to three months, the average ET of all zones continued to increase (all *P* < 0.05). Although the mean ET was decreased in all analyzed zones from three months to six months, the differences were not statistically significant (Table 3).

### 3.3. Correlation Analysis

The postoperative epithelial thickening at 6 months positively correlated with the amount of programmed SE correction in the central (*r* = 0.398, *P* = 0.001) and paracentral (*r* = 0.472, *P* < 0.001) zone; however, the correlation was opposite in the peripheral zone (*r* = −0.340, *P* = 0.006). The correlation analysis also revealed that the epithelial thickening significantly decreased with the increase in the intended diameters of the optical zone in the central (*r* = −0.289, *P* = 0.020) and paracentral (*r* = −0.339, *P* = 0.006) zones; however, the correlation was positive in the mid-peripheral zone (*r* = 0.415, *P* = 0.001) (Table 4).

Six months postoperatively, the spherical equivalent was found to be negatively associated with the postoperative epithelial thickening in the central zone (*r* = −0.313, *P* = 0.012) and with the difference of mean ET between central and paracentral zone (*r* = −0.291, *P* = 0.020). No significant correlation was observed between other ET parameters and refractive error (Table 5).

## 4. Discussion

Corneal epithelial remodeling is a crucial change induced by corneal refractive surgery. In the present study, using the wide-field OCT, we mapped the 9 mm diameter area of corneal epithelial remodeling induced by SMILE from one day to six months, postoperatively. The ET changes of different analyzed zones were shown to significantly correlate with the degree of refractive correction or with the diameter of the optical zone. Furthermore, the data demonstrated that the refractive error was related to the epithelial remodeling in central and paracentral zones.

The corneal epithelial remodeling induced by SMILE has been reported in previous studies. Vestergaard et al. and Reinstein et al. reported prominent epithelial thickening in the central cornea using OCT and very high-frequency digital ultrasound (VHFS) [22, 23], respectively. Subsequently, Luft et al. and Ganesh et al. reported the asymmetrical profile of epithelial hyperplasia within the 6 mm diameter cornea by OCT [10, 15]. Recently, Ryu et al. and Kanellopoulos et al. compared the epithelial thickness changes between SMILE and LASIK [16, 24], and their data revealed a negative meniscus-like lenticular pattern of corneal epithelial thickening after both procedures. In the present study, the mean ET of the central 2 mm increased by 4.37 *μ*m in six months postoperatively, which was in good agreement with the findings of Kanellopoulos et al. [24] and Ganesh et al. [10], although this result was lower than that reported by Luft et al. [15]. The discrepancy may be due to the different diameters of analyzed zones and the different preoperative SE of the participants. In the paracentral zone, our results demonstrated that epithelial hyperplasia is more pronounced in the temporal zone than in the nasal zone. This asymmetric profile is similar to that reported by Luft et al. [15]. Reinstein et al. and Fan et al. also reported a corresponding nonuniform epithelial thickening pattern after LASIK [8, 19]. It was assumed that this horizontal asymmetrical epithelial thickness change might result from the correction of astigmatism and the mechanical force exerted by blinking. The mid-peripheral zone in the present study was defined as the annular ring between 5 mm and 7 mm in diameter. Our analyzed area was 1 mm larger than that of the previous studies [10, 16, 24], which was from 5 mm to 6 mm in diameter. Nevertheless, in line with the previous studies [8,19], the epithelial thickening and horizontal asymmetry of ET were lower in the mid-peripheral zone than in the paracentral zone. Meanwhile, the vertical asymmetry of ET, which was considered as the influence of tear film [25,26], was simultaneously observed in paracentral and mid-peripheral zones. Overall, the present data suggest that the epithelial remodeling following the myopic SMILE was more likely to have a nonuniform lenticule-like pattern, rather than a negative meniscus-like pattern, within the 7 mm diameter of the cornea.

Merely a few studies have investigated the ET changes of the peripheral cornea after corneal refractive surgery. Using the VHFS, Reinstein et al. found that the epithelial thinning occurred in an annular area from 5.6 to 8 mm in diameter of the cornea, with no significant change from 8 mm to 10 mm diameter after myopic LASIK [23]. Fan et al. reported a significant decrease of ET in the peripheral cornea with OCT [19]. The present result was consistent with previous findings; we found that the average ET of the peripheral zone continued to decrease from the first 24 hours to one week after SMILE. This finding may be due to the intensive curvature change at the outer edge of the optical zone. Since the mean diameter of the optical zone was 6.53 ± 0.2 mm, most of the epithelial thinning induced by the curvature change occurred in the peripheral zone. From one week to three months, the ET of peripheral zone significantly increased, which was in accordance with changes of ET within 7 mm diameter cornea. We speculated that this phenomenon might be because the corneal epithelial cells are renewed and migrate centripetally from peripheral cornea.

In the present study, we assessed the longitudinal ET changes from one day to six months after SMILE. In the first week postoperatively, there was no significant change of ET within the 7 mm diameter analyzed area. In contrast, Luft et al. reported that the average ET increased with the highest rate during the first 24 hours, postoperatively [15]. Ganes et al. reported a nonsignificant overnight thinning in most analyzed sections, yet a significant epithelial thinning in the superior zone [10]. These controversial findings were also demonstrated in previous studies on myopic LASIK [8, 9]. It was speculated that this temporary fluctuation might result from the surgically induced epithelial edema and abnormal distribution of the tear film at the early stage postoperatively. Subsequently, we found a sustained increase in ET from one week to three months, which is consistent with the previous studies [10, 15, 16, 24]. Interestingly, compared with the average ET at three months, a nonsignificant epithelial thinning was observed at six months in the present study. Merely two studies reported that the SMILE induced ET changes with a follow-up period longer than three months [15, 24]. These studies also showed that there was no significant difference in ET from three months to six months.

Various studies have shown that epithelial thickening is correlated with the attempted myopic correction [9, 10, 16, 20]. In the present study, the correlation analysis also demonstrated that the epithelial hyperplasia became greater with the increase in corrected SE in the central and paracentral zones, while the correlation was opposite in the peripheral zone. This finding could be explained by the “rate of change of curvature” hypothesis. As hypothesized, a more significant curvature change induced by higher myopic correction would lead to more prominent epithelial thickening within the central optical zone. On the contrary, at the outer edge of the optical zone, a significant epithelial thinning occurred to restore the smooth corneal surface [27].

The optical zone diameter was reported to be another critical factor, which affects the ET changes after LASIK [9]. It was suggested that a larger optical zone might reduce epithelial hyperplasia [27]. For the first time, we mapped the epithelial remodeling of the entire optical zone after SMILE and found a consistent result in the central and paracentral zones. Additionally, our data showed a positive correlation between the diameter of the optical zone and the epithelial thickening in the mid-peripheral zone. A possible explanation was that the epithelial thinning at the outer edge of optical zone would be located in the peripheral zone when the optical zone was larger than 6.5 mm.

The relationship between the significant epithelial thickening and visual outcomes of the corneal refractive surgery remains inconclusive [10, 15, 16]. Cho et al. recently reported that the difference in ET between the central 2 mm and 5–6 mm diameter zones had a significant impact on the residual refractive error after LASIK [28]. Similarly, the present study demonstrated that the manifest refractive SE at six months was significantly related to the difference of average ET between the central and paracentral zones, and the epithelial thickening of central zone. Therefore, we suggested that the lenticule-like epithelial thickening profile may relate to the refractive regression after myopic SMILE procedure. Further in-depth investigations are needed to verify these findings.

There were some limitations to the present study. First, the follow-up period of six months may be inadequate to thoroughly observe the epithelial remodeling after SMILE, because a fluctuation of ET was detected at the 6-month time point. Second, dry eye examinations were not performed postoperatively. Previous studies reported that dry eyes could affect the ET [29, 30]. Therefore, assessing dry eye may reveal subtle differences. We excluded patients with preoperative moderate-to-severe dry eyes. Hence, these present results were unlikely to be significantly different, even if dry eye assessments were performed. Regardless of these, future studies with longer follow-up periods and dry eye examinations are suggested to exhaustively investigate the epithelial remodeling after SMILE.

## 5. Conclusions

The present results demonstrate an asymmetric lenticule-like pattern of epithelial remodeling over the 9 mm diameter zone of the cornea, subsequent to the myopic SMILE. The amount of myopic correction and intended diameters of the optical zone are correlated to the epithelial thickness changes, both inside and outside the optical zone. In addition, the present data also suggests that the epithelial remodeling in central and paracentral cornea may affect refractive outcomes of SMILE.

## Figures and Tables

**Table 1:** Demographic characteristics.

Characteristic	Value
Patients/eyes	64/64
Male: female	23 : 41
Mean age (years)	28.14 ± 6.38 (18 to 47)
SE (D)	−5.76 ± 2.01 (−9.88 to −1.25)
Sphere (D)	−5.39 ± 1.92 (−9.25 to −1.00)
Cylinder (D)	−0.73 ± 0.61 (−2.50 to 0)
Optical zone (mm)	6.53 ± 0.20 (6.0 to 6.9)
Central corneal thickness (*μ*m)	540.6 ± 28.9 (481 to 613)
CDVA (logMAR)	−0.12 ± 0.06 (−0.20 to 0)

D: diopters; SE: spherical equivalent; SD: standard deviation.

**Table 2:** Clinical features and refractive outcomes before and after SMILE.

	Preoperative	1 day postop	1 week postop	1 month postop	3 months postop	6 months postop
UDVA (logMAR)	1.04 ± 0.30	0.01 ± 0.12	−0.03 ± 0.07	−0.06 ± 0.07	−0.08 ± 0.09	−0.10 ± 0.08
	(0.30 to 1.52)	(−0.20 to 0.20)	(−0.20 to 0.20)	(−0.20 to 0.10)	(−0.20 to 0.1)	(−0.20 to 0)
CDVA (logMAR)	‒0.12 ± 0.06	—	—	−0.10 ± 0.05	−0.12 ± 0.06	−0.13 ± 0.07
	(‒0.20 to 0)			(−0.20 to 0.1)	(−0.20 to 0)	(−0.30 to 0)
Sphere (D)	‒5.39 ± 1.92	—	—	0.10 ± 0.19	0.03 ± 0.21	0.01 ± 0.16
	(‒9.25 to ‒1.00)			(−0.50 to 0.50)	(−0.75 to 0.50)	(−0.50 to 0.50)
Cylinder (D)	‒0.73 ± 0.61	—	—	−0.14 ± 0.20	−0.10 ± 0.20	−0.09 ± 0.21
	(‒2.50 to 0)			(−0.50 to 0)	(−0.75 to 0.50)	(−0.75 to 0.50)
SE (D)	‒5.76 ± 2.01	—	—	0.03 ± 0.22	−0.02 ± 0.25	−0.05 ± 0.22
	(‒9.88 to ‒1.25)			(−0.75 to 0.50)	(−0.75 to 0.75)	(−0.75 to 0.75)

UDVA: uncorrected distance; CDVA: corrected distance visual acuity; D: diopters; SE: spherical equivalent. Values are presented as mean ± standard deviation (range).

**Table 3:** Epithelial thickness changes in central, paracentral, mid-peripheral, and peripheral zones over time.

	Preoperative mean ± SD (*μ*m)	1 day (1) mean ± SD (*μ*m)	1 week (2) mean ± SD (*μ*m)	1 month (3) mean ± SD (*μ*m)	3 months (4) mean ± SD (*μ*m)	6 months (5) mean ± SD (*μ*m)
Central	52.33 ± 2.70	52.88 ± 3.36	52.44 ± 2.87	55.58 ± 3.67	57.39 ± 3.67	56.70 ± 3.22
*P* value		1.000^a^	1.000^a^ 1.000^b^	<0.001^a^ <0.001^c^	<0.001^a^ 0.003^d^	<0.001^a^ 1.000^e^
Paracentral	52.23 ± 2.45	53.23 ± 3.13	53.01 ± 2.74	55.87 ± 2.83	57.50 ± 2.95	56.59 ± 2.82
*P* value		0.240^a^	1.000^a^ 1.000^b^	<0.001^a^ <0.001^c^	<0.001^a^ 0.003^d^	<0.001^a^ 0.533^e^
Mid-peripheral	51.75 ± 2.31	52.09 ± 3.00	51.25 ± 2.46	53.17 ± 2.48	54.34 ± 2.50	53.36 ± 2.36
*P* value		0.930^a^	1.000^a^ 0.069^b^	0.001^a^ <0.001^c^	<0.001^a^ 0.002^d^	<0.001^a^ 0.131^e^
Peripheral	50.03 ± 2.39	48.41 ± 2.80	46.77 ± 2.29	48.33 ± 3.08	49.38 ± 3.26	48.46 ± 2.63
*P* value		<0.001^a^	<0.001^a^ <0.001^b^	<0.001^a^ <0.001^c^	0.791^a^ 0.025^d^	<0.001^a^ 0.086^e^

SD: standard deviation. ^a^Comparison from preoperative values; ^b^comparison between (1) and (2); ^c^comparison between (2) and (3); ^d^comparison between (3) and (4);^e^comparison between (4) and (5).

**Table 4:** Correlation between epithelial thickening and factors at six months postoperatively.

Parameter		Central zone	Paracentral zone	Mid-peripheral zone	Peripheral zone
SE of correction (D)	*P*	0.001	<0.001	0.163	0.006
	*r*	0.398	0.472	0.177	−0.340
OZ (mm)	*P*	0.020	0.006	0.001	0.189
	*r*	−0.289	−0.339	0.415	0.166

SE: spherical equivalent; OZ: optical zone.

**Table 5:** Correlation between spherical equivalent and corneal epithelial thickness parameters at six months postoperatively.

Parameter (*μ*m)	*r*	*P*
ET of central zone	−0.231	0.066
ET of paracentral zone	−0.127	0.316
ET of mid-peripheral zone	0.023	0.859
Epithelial thickening of central zone	−0.313	0.012
Epithelial thickening of paracentral zone	−0.073	0.569
Epithelial thickening of mid-peripheral zone	0.081	0.523
Difference of mean ET between central and paracentral zone	−0.291	0.020
Difference of mean ET between central and mid-peripheral zone	0.218	0.084

ET: epithelial thickness.

## Data Availability

The datasets used in the current study are available from the corresponding author upon reasonable request.
